# Transcranial direct current stimulation to improve cognitive function in patients with vascular cognitive impairment: a literature review

**DOI:** 10.3389/fnagi.2026.1829594

**Published:** 2026-07-01

**Authors:** Jiamian Shi, Xiaodong Li, Yubiao Sun, Ling Guan

**Affiliations:** 1 Department of Biomedical Engineering, Medical Science and Engineering, Beijing Institute of Technology, Beijing, China; 2 School of Interdisciplinary Science, Beijing Institute of Technology, Beijing, China; 3 Department of Neurology, Beijing Tiantan Hospital, Capital Medical University, Beijing, China

**Keywords:** cerebral small vessel disease, noninvasive neuromodulation, stroke, transcranial direct current stimulation, vascular cognitive impairment

## Abstract

Vascular pathology represents the second most common cause of cognitive impairment after Alzheimer’s disease and contributes substantially to mixed dementia involving both vascular and neurodegenerative mechanisms. Vascular cognitive impairment (VCI) emerges as a term encompassing cognitive impairments resulting from stroke, cerebral small vessel disease (CSVD), or other forms of vascular pathology. While numerous studies have demonstrated the beneficial effects of transcranial direct current stimulation (tDCS) on mild cognitive impairment and dementia, evidence specifically addressing its efficacy in VCI remains limited. We conducted a structured narrative review to summarize the effects of tDCS on cognitive rehabilitation in individuals with VCI. Studies were included if participants had cognitive impairment related to vascular pathology and if cognitive severity ranged from mild cognitive impairment to dementia. Eighteen studies met the inclusion criteria. Among the included studies, 15 were conducted in post-stroke cognitive impairment (PSCI) populations, whereas only three studies focused on non-stroke VCI. Across the included studies, tDCS has demonstrated promising effects on global cognition and executive function in PSCI populations. Combining tDCS with cognitive or motor rehabilitation appeared to further facilitate cognitive recovery in PSCI populations. Neuroimaging and EEG findings suggest that tDCS may be associated with cognitive improvement through potential modulation of cortical excitability, neural oscillatory activity, and functional connectivity. In addition, preliminary evidence suggests tDCS may have the potential to modulate cerebral blood flow, indicating a potential modulatory effect on neurovascular coupling. However, most available studies were conducted in patients with PSCI, with insufficient evidence in non-stroke VCI populations related to CSVD. Furthermore, the neurophysiological mechanisms by which tDCS modulates vascular and cognitive function remain poorly understood, warranting further investigation in future studies.

## 1 Introduction

### 1.1 Vascular cognitive impairment

Vascular cognitive impairment (VCI) is an umbrella term encompassing all degrees of cognitive impairment attributed to vascular brain pathology, ranging from subjective cognitive decline (SCD) to mild VCI, and ultimately to vascular dementia (VaD) (van der Flier et al., 2018). VaD is recognized as the second most prevalent cause of cognitive decline after Alzheimer’s disease (AD), with patients exhibiting a shorter average survival time than those with AD (O'Brien and Thomas, 2015; Staekenborg et al., 2016).

VCI may arise solely from vascular pathology or result from a combination of vascular and AD pathology. This co-occurrence is often referred to as mixed dementia, in which vascular and neurodegenerative pathologies, such as AD or dementia with Lewy bodies (DLB), jointly contribute to cognitive impairment. Previous studies indicate that concomitant AD pathology is present in approximately 77% of patients with VaD, whereas dementia resulting exclusively from pure vascular pathology is relatively uncommon (Barker et al., 2002; Schneider et al., 2007). The vascular pathologies of VCI may include ischemic and hemorrhagic stroke (Makin et al., 2013; Weaver et al., 2021), as well as cerebral small vessel disease (CSVD) (Hamilton et al., 2020; Yang et al., 2022b). Post-stroke cognitive impairment (PSCI) refers to cognitive impairment attributable to cerebrovascular injury following stroke. According to the Vascular Impairment of Cognition Classification Consensus Study (VICCCS), post-stroke dementia is classified as a subtype of major VCI, while the classification of mild VCI subtypes remains less clearly defined (Skrobot et al., 2016). Accordingly, PSCI is generally considered a subtype of VCI, although the extent to which individual studies meet standardized VCI criteria varies. CSVD refers to all the pathological processes that affect the small arteries, arterioles, capillaries, and small veins in the brain (Pantoni, 2010). According to the STandards for ReportIng Vascular changes on nEuroimaging (STRIVE) criteria (Wardlaw et al., 2013; Duering et al., 2023), common neuroimaging features of CSVD visible on MRI or CT include recent small subcortical infarcts, lacunes, white matter hyperintensities (WMH), perivascular spaces, microbleeds, and brain atrophy. Evidence suggests that WMH, lacunes, microbleeds, and perivascular space dilation are correlated with VCI (Liu, 2014; Shi et al., 2023; Filler et al., 2024).

Compared to AD which primarily impairs memory function, VCI caused by vascular pathology results in diverse syndromes, partly depending on the type and the location of the lesions (O'Brien et al., 2003). Consequently, the Montreal Cognitive Assessment (MoCA), which includes a broader range of subtasks encompassing executive function and is more sensitive to subtle cognitive decline, is often preferred over the Mini-Mental State Examination (MMSE) (van der Flier et al., 2018).

The diagnosis of cognitive impairment is based on the decline in one or more of the five core cognitive domains: executive function, attention, memory, language, and visuospatial function (Skrobot et al., 2018). Supporting neuroimaging evidence demonstrating the correlation between cognitive impairment and vascular disease is essential. According to VICCCS, mild VCI requires impairment in at least one cognitive domain, with mild or no impairment in instrumental activities of daily living (IADLs) (Skrobot et al., 2016). Major VCI or VaD requires clinically significant deficits of sufficient severity in at least one cognitive domain and severe disruption to Activities of Daily Living or Instrumental Activities of Daily Living (ADLs/IADLs) (Skrobot et al., 2016).

Currently, the management of VCI relies on cognitive training and lifestyle interventions. While these approaches can be beneficial, they exhibit a slow onset of improvement and limited individualization to address the specific needs of individual patients.

### 1.2 Transcranial direct current stimulation

Recently, there has been increasing interest in the potential application of transcranial direct current stimulation (tDCS) to enhance cognitive function (Harris, 2023). tDCS belongs to non-invasive neuromodulation techniques and makes use of direct current to modulate spontaneous cortical activity and to promote neuroplasticity (Paulus, 2011). Because of its relatively low cost, portability and ease of application, tDCS has been increasingly explored as an adjunctive intervention in cognitive impairment and neurological rehabilitation.

In a typical tDCS protocol, weak direct current is applied to the scalp through two or more electrodes, with the anodal stimulation generally associated with increased excitability, and cathodal stimulation associated with reduced excitability (Stagg and Nitsche, 2011). The current intensity is usually weak (1–2 mA), which is insufficient to depolarize the membrane potential of neurons to evoke a spike (Miniussi et al., 2013). Nevertheless, it is believed that the mild stimulation from the anode can induce a subthreshold depolarization of neuronal membranes, thereby increasing neuronal excitability (Purpura and McMurtry, 1965). Conversely, the cathodal tDCS is thought to deepen the resting membrane potential, making neurons less likely to depolarize and thus reducing excitability. Research has shown that tDCS can effectively modulate cognition (Shin et al., 2015), and a meta-analysis revealed that small but statistically significant effects of tDCS on working memory in patients with various neurological disorders (Begemann et al., 2020).

There are multiple mechanisms that may explain the cognitive benefits of tDCS. At the cellular level, tDCS may alter neuronal excitability and synaptic efficacy (Stagg and Nitsche, 2011). At the network level, it may modulate functional connectivity and the efficiency of large-scale cognitive networks (Peña-Gómez et al., 2012; Kunze et al., 2016). In addition, tDCS has been proposed to enhance the effects of cognitive rehabilitation by promoting neuroplasticity (Podda et al., 2016; Siegert et al., 2021). Neuroplasticity is an intrinsic property of the nervous system that enables the brain to reorganize and adapt in response to environmental changes or injury (Pascual-Leone et al., 2005). By reducing neuronal activation thresholds and strengthening synaptic efficacy, tDCS may create a more favorable neural environment to prepare neurons for training, promoting neuroplasticity and facilitating learning processes (Simonsmeier et al., 2018; Yavari et al., 2018).

Although several reviews have examined the effects of tDCS in cognitive impairment more broadly, evidence specifically focusing on tDCS for cognitive rehabilitation in VCI remains limited. Therefore, the present review aims to summarize clinical studies employing tDCS for cognitive rehabilitation in patients with VCI, clarify the current status and limitations of the evidence, and discuss future directions for developing stimulation strategies for this population.

## 2 Materials and methods

### 2.1 Search strategy

This review was conducted as a narrative review using a structured literature search of PubMed, Web of Science (WOS), and Scopus databases, and relevant studies published up to 4 June 2026 were identified using predefined keywords. Only English-language articles were included.

The search strategy combined keywords related to tDCS and VCI (“PSCI,” “VCI,” “mixed dementia”). Given that PSCI is generally regarded as a subtype of VCI due to its vascular etiology, PSCI-related terms were also included in the search strategy to ensure comprehensive identification of relevant studies. Mixed dementia refers to the coexistence of vascular and neurodegenerative pathologies contributing to cognitive impairment. Studies involving mixed dementia were included only when vascular contributions to cognitive impairment were explicitly described or considered a primary component.

The final search was conducted in June 2026. And the search formula in PubMed was (“tDCS” AND (“VCI” OR “PSCI” OR “mixed dementia”) NOT “Review” NOT (“animal” NOT “Humans”)). The search syntax was adapted for WOS and Scopus according to their respective indexing systems while maintaining the same conceptual search framework. The complete database-specific search strategies are provided in Supplementary Table S1.

### 2.2 Inclusion and exclusion criteria

#### 2.2.1 Population

Patients were eligible if they were aged 18 years or older and had cognitive impairment attributable to vascular pathology, preferably defined according to the VICCCS framework. For studies conducted before the development or widespread adoption of VICCCS, earlier established diagnostic criteria, such as NINDS-AIREN, were accepted when the study population represented cognitive impairment attributable to vascular pathology. All of the following criteria were required:

1. Cognitive impairment ranging from mild VCI to VaD;
2. Vascular pathology underlying the cognitive impairment, including stroke or CSVD;
3. Impairment in at least one cognitive domain, including executive function, attention, memory, or information processing.

Studies focusing exclusively on the recovery from aphasia or visuospatial neglect were excluded.

#### 2.2.2 Intervention

tDCS was required to be the primary intervention; no restrictions were applied regarding stimulation parameters or stimulation sites.

#### 2.2.3 Study design

All types of clinical studies were considered, including randomized controlled trials (RCTs), cross-over studies and case reports.

#### 2.2.4 Outcome

The primary or secondary outcome measures were required to include neuropsychological assessments of global cognition, attention, memory, executive function, language, or visuospatial abilities. Studies that relied solely on task-based behavioral paradigms for cognitive evaluation were excluded. Both baseline and post-treatment measurements were required for inclusion.

### 2.3 Selection process

Studies were initially identified from each database according to the predefined search strategy, and duplicate records were removed prior to screening. Title and abstract screening, as well as full-text eligibility assessment, were independently conducted by two reviewers (JS and XL). Titles and abstracts were first screened to exclude articles that did not meet the predefined inclusion criteria. Any disagreements regarding study selection were resolved through discussion and, when necessary, consultation with a third reviewer (LG) to achieve consensus. Detailed characteristics of the included studies are summarized in Tables 1, 2.

**Table 1:** Summary of clinical studies investigating tDCS for cognitive rehabilitation in patients with PSCI.

Author, year	Group and task	Stimulation target	Stimulation parameters	Reported outcome
Park et al. (2013)	*n* = 6 tDCS + CACR *n* = 5 sham-tDCS + CACR (tDCS and cognitive training are concurrent)	Anode: F3 and F4 Cathode: non-dominant arm	2 mA, 30 min per session, 5 sessions per week, 18.5 days (tDCS group)/17.8 days (control group) on average.	tDCS showed significant improvement on post/pre ratio of scores on the auditory and visual continuous performance test.
Leo et al. (2016)	*n* = 1. Task: 3 protocols in sequential: (a) traditional CT, (b) CCT, and (c) CCT combined with tDCS. A 2-week interval separated each session.	Cathode: T8 Reference: left shoulder	1 mA, delivered for 15 min for two sessions separated by 5 min, five times per week for 8 weeks.	The patient showed the best neuropsychological improvement in attentional and language domains after the third training.
Ko et al. (2022)	*n* = 12 remotely supervised tDCS + CCT *n* = 14 sham-tDCS + CCT (concurrent)	Anode: F3 Cathode: right supraorbital region	2 mA, 30 min per session, 5 sessions per week, 4 weeks.	The real group showed significant improvement in K-MoCA, particularly in patients with lower baseline K-MoCA or with left hemispheric lesions. And the adherence rate of tDCS was 98.4%.
Shaker et al. (2018)	*n* = 20 tDCS + CCT *n* = 20 sham-tDCS + CCT (not concurrent)	Anode: F3 and F4 Cathode: contralateral supraorbital area	2 mA, 30 min per session, three sessions per week (every other day) for 1 month.	Both groups showed significant improvements in attention and concentration, figural memory, and functional independence measure, while the real stimulation group received more significant improvement.
Chen et al. (2024)	*n* = 18 conventional CT *n* = 18 tDCS *n* = 18 CACT *n* = 18 CACT + tDCS (concurrent)	Anode: F3 Cathode: right supraorbital region	2 mA, 20 min per session, 5 sessions per week, 3 weeks.	CACT + tDCS group improved the most in MoCA and IADL scores, especially in visuospatial and executive domains. Only CACT + tDCS group improved significantly in breath holding index.
De Luca et al. (2025)	*n* = 10 VR + tDCS *n* = 10 VR + sham-tDCS (not concurrent)	Anode: F7 Cathode: right supraorbital region	2 mA, 20 min per session, 5 sessions per week, 4 weeks.	The real stimulation group improved significantly in MMSE scores and showed greater gain in executive function, while also showed enhancement of cognitive processing speed (reduced P300 latency).
Kazinczi et al. (2025)	*n* = 18 tDCS *n* = 18 sham-tDCS + inhibitory control training (ICCT) *n* = 18 sham-tDCS + ICCT	Anode: AFz Cathode: Pz	2 mA, 12 min per session, 5 sessions per week, 2 weeks.	The results supported the combined application of stimulation and cognitive training on working memory, while no significant improvement was shown in groups with any single training.
Yun et al. (2015)	*n* = 15 left tDCS *n* = 15 right tDCS *n* = 15 sham stimulation	Anode: T3 (left group)/T4 (right group) Cathode: not reported	2 mA, 30 min per session, 5 sessions per week, 3 weeks.	The left tDCS group improved significantly in the memory function but not in global cognition or daily living functionality.
Ai et al. (2024)	*n* = 38 tDCS *n* = 38 sham-tDCS	Anode: F3 Cathode: right supraorbital region	2 mA, 30 min per session, 5 sessions per week, 2 weeks.	tDCS group improved significantly in general cognitive function and working memory, which showed interaction with COMT genotype.
Yang et al. (2022a)	*n* = 22 PSCI *n* = 14 healthy individuals	Anode: F3 Cathode: F4	2 mA, 30 min per session, 7 sessions per week, 2 weeks.	Patients with stroke increased significantly in MoCA and MMSE scores after 7 times and 14 times tDCS. fNIR results supported the cortical activation in the left superior temporal cortex and the VLPFC after tDCS.
Hu et al. (2023)	Total n = 34 *n* = 12: sham stimulation *n* = 12: rTMS *n* = 10 (2 drop-out): simultaneous rTMS + tDCS (all groups receive non-concurrent cognitive training)	rTMS: F3 tDCS Anode: T5/T6 of the affected side tDCS Cathode: P3/P4 of the unaffected side	rTMS: 5 Hz, each cycle lasted for 5 s with a 25 s interval, 20 min per session, 5 sessions per week, 4 weeks. tDCS: 1.2 mA, 20 min per session, 5 sessions per week, 4 weeks.	Delayed recall in MoCA and delayed processing in RBMT scored better in the rTMS-tDCS combination group, with MMN and P300 latency significantly shorter.
Chu et al. (2022)	*n* = 21 iTBS + CT *n* = 19 tDCS + CT *n* = 20 CT (not concurrent)	iTBS: F3 tDCS Anode: F3 tDCS Cathode: right shoulder	iTBS: triplet 50 Hz bursts, repeated at 5 Hz for 20 times, a total duration of 3 min 20 s, 5 sessions per week, 6 weeks. tDCS: 2 mA, 20 min per session, 5 sessions per week, 6 weeks.	All groups improved significantly in cognitive function, with the stimulation groups showing greater improvement. fMRI results related the cognitive improvement from tDCS to the activation of the frontopolar cortex, while iTBS to the activation of the stimulation site and some distant regions.
Zhang et al. (2024)	*n* = 30 tDCS *n* = 30 motor-cognitive intervention *n* = 30 tDCS + motor cognitive intervention (non-concurrent)	Anode: F3 Cathode: right supraorbital region	2 mA, 20 min per session, 5 sessions per week, 4 weeks.	In the combination group, total and subtest scores of MoCA improved significantly except for attention, and LOTCA improved significantly except for motor praxis.
Kim et al. (2019)	*n* = 11 tDCS + Nintendo Switch *n* = 10 sham tDCS + Nintendo Switch *n* = 9 tDCS-only group (not concurrent)	Fixed to stimulate the affected side of motor cortex in the vertical direction from the ear	Intensity not reported, 20 min per session, 2 sessions per week, 8 weeks.	The tDCS + Nintendo Switch game group showed significant higher improvement in cognition and hand function compared to the other groups.
Wang et al. (2022)	*n* = 12 tDCS + CT *n* = 12 sham-tDCS + CT (not concurrent)	Anode: F3 Cathode: Fp2	2 mA, 20 min per session, 7 sessions per week, 1 week.	The real stimulation group improved significantly in the executive function. And EEG results suggested that the improvement was related to the enhancement of theta power in the affected region.

CT, cognitive training; CCT, computerized cognitive training; CACR, computer-assisted cognitive rehabilitation; VR, virtual reality; DLPFC, dorsolateral prefrontal cortex; K-MoCA, Korean version of the Montreal Cognitive Assessment; K-MMSE, Korean version of the Mini-Mental State Examination; IADL, instrumental activities of daily living; RBMT, Rivermead Behavioral Memory Test; LOTCA, Loewenstein Occupational Therapy Cognitive Assessment; MBI, Modified Barthel Index.

**Table 2:** Summary of clinical studies investigating tDCS for cognitive rehabilitation in patients with non-stroke VCI.

Author, year	Group and task	Stimulation target	Stimulation parameters	Reported outcome
André et al. (2016)	*n* = 13 tDCS *n* = 8 sham-tDCS	Anode: F3 Cathode: right supraorbital region	2 mA, 20 min per session, 4 successive sessions, at-home stimulation	Only the real stimulation group improved in visual short-term memory, verbal working memory, and executive control.
Gangemi et al. (2024)	*n* = 15 tDCS *n* = 15 sham-tDCS	Anode: F7 Cathode: right supraorbital area	2 mA, 20 min per session, 7 sessions per week, 2 weeks	EEG results indicated that tDCS led to reduced P300 latency and increased P300 amplitude. Significant improvements in MMSE scores were also observed compared with the control group.
Cao et al. (2026)	*n* = 20 HD-tDCS	Central electrode: F3 Peripheral electrode: Fp1, Fz, C3, F7	2 mA, 20 min per session, 7 sessions per week, 2 weeks	Improvements in global cognition, memory, information-processing speed, and attention, accompanied by decreased functional connectivity between the hippocampal cognitive subregion and the left dorsolateral superior frontal gyrus.

DLPFC, dorsolateral prefrontal cortex; MMSE, Mini-Mental State Examination.

## 3 Results

**Figure 1:**
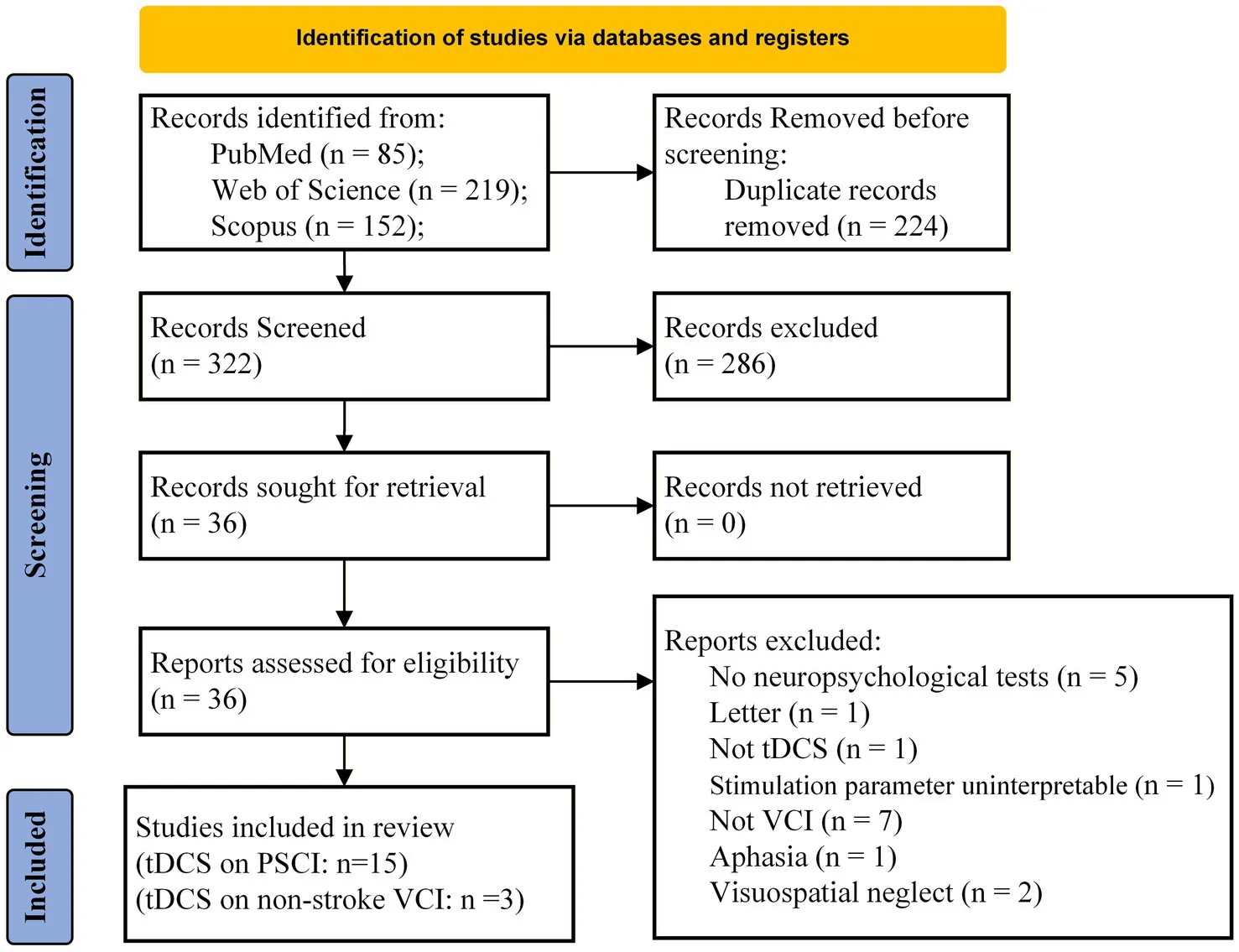
Study selection process.

**Figure 2:**
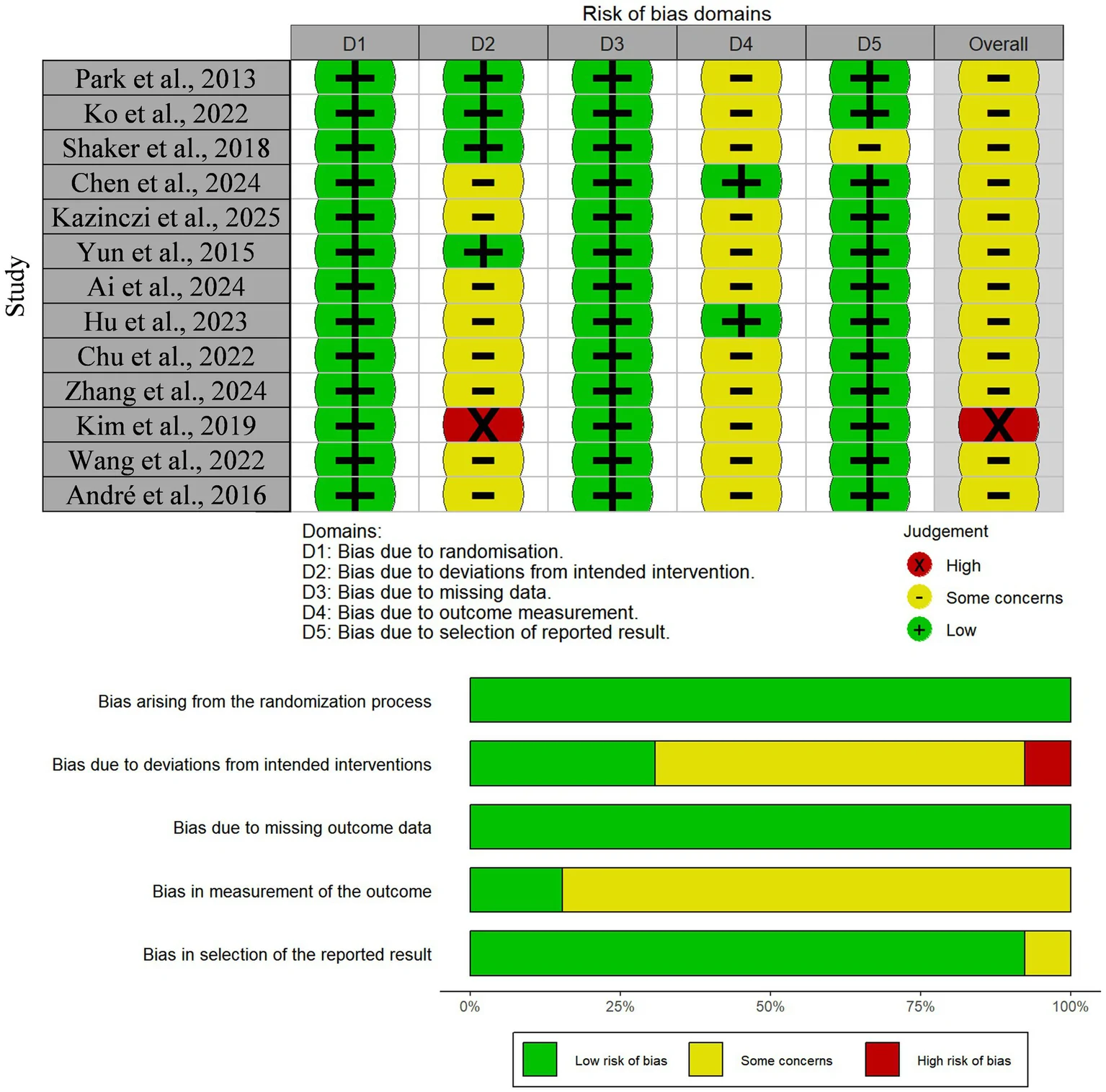
Risk of bias of included RCT studies.

Figure 1 summarizes the study selection process at each stage. Following identification, a total of 322 articles were identified and screened. After title/abstract screening and full-text eligibility assessment, 15 studies investigating the effects of tDCS on PSCI, and three studies examining the therapeutic effects of tDCS on non-stroke VCI were included. Among the 18 included studies, one was a case study (Leo et al., 2016), three were controlled trials without formal randomization (Yang et al., 2022a; Gangemi et al., 2024; De Luca et al., 2025), one was an open-label study (Cao et al., 2026), and the remaining 13 were RCTs. Non-randomized, uncontrolled, and case studies were not formally assessed using RoB 2 and were summarized narratively. The Cochrane Risk of Bias 2 (RoB 2) assessment of the 13 RCT studies showed that concerns were mainly concentrated in the domains of outcome measurement and deviations from intended interventions, as shown in Figure 2. These concerns were largely related to unclear reporting of outcome assessor blinding, and the use of single-blind rather than double-blind protocols.

### 3.1 tDCS to modulate cognitive functions in PSCI

In total, 15 articles meeting the inclusion criteria for using tDCS to modulate cognitive function in PSCI were included and summarized in Table 1. The following sections analyze the effects of tDCS on cognition, with studies categorized according to whether tDCS was combined with diverse complementary interventions focused on specific cognitive domains. These categories do not represent entirely distinct topics but rather offer diverse perspectives on the effects of tDCS. By organizing and comparing these studies, a comprehensive understanding of the impact of tDCS on post-stroke cognitive rehabilitation can be established. However, the included studies exhibited substantial heterogeneity in terms of stimulation parameters (e.g., target regions, intensity, and duration), patient characteristics, and cognitive outcome measures, which should be considered when interpreting the findings.

#### 3.1.1 tDCS in combination with cognitive rehabilitation on cognition

Across the included studies, several articles suggested that combining tDCS with concurrent cognitive training may produce greater improvements in cognition compared with administering tDCS or cognitive training alone. Cognitive trainings approaches included traditional cognitive training (Chu et al., 2022; Wang et al., 2022; Kazinczi et al., 2025), computer-assisted cognitive rehabilitation (CACR) or computerized cognitive training (CCT) (Park et al., 2013; Leo et al., 2016; Shaker et al., 2018; Ko et al., 2022; Chen et al., 2024), and virtual reality (VR) based cognitive training (De Luca et al., 2025). This synergistic effect was evidenced by the improvement of global cognitive measures, such as larger gains in MMSE or MoCA scores when stimulation was paired with cognitive training tasks (Ko et al., 2022; Yang et al., 2022a; Ai et al., 2024; Chen et al., 2024; De Luca et al., 2025). As for specific cognitive domains, tDCS also showed positive effects on attention (Leo et al., 2016; Shaker et al., 2018), working memory (Kazinczi et al., 2025), and executive function (Yang et al., 2022a; Chen et al., 2024; De Luca et al., 2025).

For example, Ko et al. (2022) evaluated the cognitive improvement and feasibility of remotely supervised tDCS in patients with chronic stroke and cognitive impairment. Anodal stimulation was performed on the left dorsolateral prefrontal cortex (DLPFC), and concurrent CCT was required in both the real stimulation and sham stimulation groups. After 20 sessions of training, the real stimulation group showed significant improvement in Korean version of the MoCA (K-MoCA), particularly among patients with lower baseline K-MoCA or with left hemispheric lesions.

Another study by Chen et al. (2024) reported that the CACT combined with tDCS group showed greater improvement in MoCA scores compared with other groups with any single intervention, especially in terms of visuospatial and executive. To assess the effects of stimulation on cerebrovascular function, Transcranial Doppler ultrasound (TCD) was used to measure changes in middle cerebral artery blood flow. The results indicated that only the CACT combined with tDCS group exhibited a significant improvement in the breath holding index (BHI) after training.

Also, there were several studies that did not introduce any cognitive training but examined the effect of tDCS alone on cognition (Yun et al., 2015; Yang et al., 2022a; Ai et al., 2024), in which the study of Yun et al. (2015) found no significant difference on the Korean version of the MMSE or the Modified Barthel Index (MBI) in any group; however, the verbal learning test on the Computerized Neuropsychological Test (CNT) revealed significant improvement in the left anodal stimulation group.

Ai et al. (2024) examined the interaction effects of COMT and BDNF genotypes on the cognitive rehabilitation. PSCI patients with or without COMT Val158Met (rs4680) and BDNF Val66Met (rs6252) SNPs were included, as determined from blood samples. The COMT Val158Met variation affects dopamine metabolism in the prefrontal cortex, influencing the effects of tDCS on working memory and executive function (Wiegand et al., 2016; Jongkees et al., 2019). The results indicated significant improvements in MoCA and backward digit span test (BDST) score in the tDCS group compared to sham. Notably, cognitive enhancements following tDCS were influenced by the COMT genotype, whereas BDNF showed no significant interaction effects.

#### 3.1.2 tDCS effects on neuroimaging and EEG

In addition to neuropsychological assessments, numerous studies have employed functional neuroimaging and EEG to explore the potential neuronal correlates of electrical stimulation (Chu et al., 2022; Wang et al., 2022; Yang et al., 2022a; Hu et al., 2023; De Luca et al., 2025). These approaches may provide complementary and objective insights into the underlying neural changes associated with the stimulation, although the evidence remains preliminary.

For example, Yang et al. (2022a) assessed cortical activation during a verbal fluency task (VFT) using functional near-infrared spectroscopy (fNIRS) in patients with stroke. Increased cortical activation was observed in the left superior temporal cortex (lSTC) after tDCS. Functional connectivity (FC) between the cerebral hemispheres of patients with stroke was lower than that of healthy controls but increased following tDCS. These findings suggest that tDCS may be associated with changes in brain network activity.

A pilot study by De Luca et al. (2025) investigated the combined effects of tDCS and VR-based cognitive training in patients with chronic ischemic stroke. In addition to greater improvements in global cognition and executive function, patients in the active tDCS group exhibited reduced P300 latency in EEG, indicating faster emergence of the P300 component of the event-related potential (ERP) following stimulus presentation. The reduction in P300 latency reflects enhanced cognitive processing efficiency, suggesting that tDCS may potentiate the cognitive benefits of immersive rehabilitation by facilitating neuroplasticity.

#### 3.1.3 tDCS in the context of multimodal neuromodulation

Over the past few years, studies have investigated the use of non-invasive brain stimulation (NIBS) methods for cognitive rehabilitation. Repetitive transcranial magnetic stimulation (rTMS) modulates neural activity by delivering short, repeated high intensity pulsed magnetic fields precisely to the stimulation point, inducing neuronal hyperpolarization or depolarization (Lioumis and Rosanova, 2022). TMS has been shown to improve global cognitive abilities and activities of daily living in stroke patients, which is correlated to increased functional connectivity of the left medial prefrontal cortex, right medial prefrontal cortex and right ventral anterior cingulate cortex (Yin et al., 2020; Li and Xiao, 2024; Zhu et al., 2024).

Hu et al. (2023) were the first to explore the bimodal neuromodulatory effects of rTMS combined with tDCS in patients with post-stroke dysmnesia. In the rTMS-tDCS group, rTMS was targeted at the left DLPFC (F3), while simultaneous tDCS was applied to the temporal lobe of the affected hemisphere. Results showed that delayed recall scores on the MoCA and delayed processing assessed by the Rivermead Behavioral Memory Test (RBMT) improved more in the rTMS-tDCS group than in the rTMS-only group. Mismatch Negativity (MMN) and P300 latency detected in EEG were significantly shorter in the rTMS-tDCS combination group, suggesting that combining rTMS and tDCS may achieve enhanced cognitive effects. However, the use of combined TMS and tDCS interventions in this study limits the ability to analyze the independent contribution of tDCS to cognitive outcomes.

Intermittent Theta-burst stimulation (iTBS) is a specific mode of rTMS in which 2-s TBS sequences (10 TBS bursts) are delivered every 10 s. Evidence indicates that TBS can enhance cortical excitability more effectively by matching the theta rhythm of natural discharge in the brain (Grossheinrich et al., 2009; Wischnewski and Schutter, 2015). Chu et al. (2022) investigated the efficacy of iTBS and tDCS combined with cognitive training for the treatment of PSCI. After 30 sessions, patients in the iTBS and tDCS groups showed greater cognitive improvement compared with those receiving only cognitive training. fNIRS results from 14 patients indicated that the cognitive improvement induced by tDCS was related to the activation of the frontopolar cortex (FPC), whereas cognitive gains from iTBS were related to activation at the stimulation site as well as distant regions.

#### 3.1.4 tDCS combined with motor rehabilitation on cognition

In recent years, tDCS has shown promising results for motor rehabilitation in post-stroke patients with dysphagia and upper limb motor impairment (Anwer et al., 2022; Gómez-García et al., 2023). Increasing evidence suggests that cognitive and motor rehabilitation are not independent but mutually influence each other (Mane et al., 2020). Accordingly, several studies have combined motor tasks with tDCS to investigate both motor and cognitive benefits.

Zhang et al. (2024) investigated the efficacy of tDCS combined with motor-cognitive intervention for PSCI in a double-blinded RCT. Patients were assigned to three groups: a tDCS group, a motor-cognitive intervention group, and a combination group. All groups showed significant improvement on the MoCA and Loewenstein Occupational Therapy Cognitive Assessment (LOTCA) scores. The combination group demonstrated greater cognitive improvements than the other two groups, except for the attention domain in the MoCA.

Kim et al. (2019) examined the combined effects of tDCS and Nintendo Switch gaming on cognitive motor learning and hand function in stroke survivors. The Nintendo Switch game is a virtual reality-based intervention played with a recognition controller on a television screen, encompassing activities of daily living, cognitive training, rhythm training, and aerobic training. After 2 sessions per week for 8 weeks, the tDCS plus game training group showed significant improvements on the Trail Making Test and grip strength compared to both the sham stimulation group and the tDCS only group, suggesting that tDCS combined with motor learning tasks can support the restoration of both cognitive and physical functions.

#### 3.1.5 tDCS on post-stroke executive dysfunction

As VCI progresses, patients exhibit diverse symptoms of cognitive impairments. Compared to patients with AD, those with VaD have been shown to perform better in verbal long-term memory but exhibit more significant impairments in frontal executive functions (Looi and Sachdev, 1999). Executive function encompasses several subdomains such as inhibition, working memory, cognitive flexibility (Sachdev et al., 2004), all of which are correlated with the frontal lobe and interact synergistically to support task performance.

Wang et al. (2022) conducted a pilot randomized controlled study to investigate the effects of tDCS on executive function and resting-state EEG in patients with post-stroke executive impairment. In the real stimulation group, 2 mA anodal tDCS was delivered for 20 min per session across seven consecutive days, while the control group received sham stimulation. The experimental group exhibited significant improvements in MoCA, Symbol Digit Modalities Test (SDMT), TMT-A, TMT-B, and Digit Span Test scores compared to controls. Resting-state EEG analysis revealed a significant post-stimulation increase in theta band power in the left central region, which was positively correlated with SDMT scores.

Finally, to facilitate comparison across studies and support the interpretation of the findings, we summarized the methodological characteristics and key limitations of studies investigating tDCS in PSCI in Table 3. As small sample sizes were a common feature across the included studies, they were not specifically listed as a study-specific limitation in the table.

**Table 3:** Study characteristics and key limitations of included studies on tDCS in PSCI.

Author, year	Study design	Sample size	Blinding	Control	Key limitations
Park et al. (2013)	Pilot RCT	*n* = 11 (2 groups: 6/5)	Double-blind	Sham	Very small sample size
Leo et al. (2016)	Case study	*n* = 1	/	/	Very small sample size; no control
Ko et al. (2022)	Pilot RCT	*n* = 26 (2 groups: 13/13)	Double-blind	Sham	Lesion heterogeneity; unclear contribution of CCT
Shaker et al. (2018)	RCT	*n* = 40 (2 groups: 20/20)	Single-blind	Sham	Only male participants included
Chen et al. (2024)	RCT	*n* = 72 (4 groups: 18/18/18/18)	Single-blind	Active controls (CCT and CACT)	Lack of sham control
De Luca et al. (2025)	Pilot quasi-randomized controlled trial	*n* = 20 (2 groups: 10/10)	Double-blind	Sham	Lack of adequate randomization
Kazinczi et al. (2025)	RCT	*n* = 54 (3 groups: 18/18/18)	Single-blind	Sham	limited cognitive outcome domains
Yun et al. (2015)	RCT^*^	*n* = 45 (3 groups: 15/15/15)	Double-blind	Sham	Depression not controlled
Ai et al. (2024)	RCT	*n* = 76 (2 groups: 38/38)	Double-blind	Sham	Small sample for EEG and fNIRS data
Yang et al. (2022a)	Controlled trial (randomization not reported)	*n* = 36 (2 groups: 22/14)	Not reported	Healthy control	Unclear randomization and blinding; no sham control
Hu et al. (2023)	Pilot RCT	*n* = 34 (3 groups: 12/12/10)	Single-blind	Sham	No tDCS-only arm
Chu et al. (2022)	RCT	*n* = 60 (3 groups: 21/19/20)	Single-blind	Active control (iTBS)	No sham control; limited cognitive outcome domains
Zhang et al. (2024)	RCT	*n* = 90 (3 groups: 30/30/30)	Double-blind	Active control	No sham control
Kim et al. (2019)	RCT	*n* = 30 (3 groups: 11/10/9)	Not reported	Sham	blinding not reported
Wang et al. (2022)	Pilot RCT	*n* = 24 (2 groups: 12/12)	Single-blind	Sham	Short intervention duration

^*^Described as a “randomized case-control study” in the original article, but classified as a RCT based on the reported random allocation and intervention design.

### 3.2 tDCS to modulate cognitive functions in non-stroke VCI

Currently, we identified only three tDCS studies specifically targeting non-stroke VCI, as shown in Table 2. Two studies enrolled patients classified as having VaD based on the severity of cognitive impairment, whereas one recent study specifically focused on patients with CSVD. The available evidence remains too limited to determine whether non-stroke VCI patients respond differently to tDCS compared with those with PSCI.

In a single-blinded randomized study conducted by André et al. (2016), 21 patients with VaD were recruited and classified as probable or possible VaD, according to international diagnostic criteria including neuroimaging data (Erkinjuntti, 1994). Three of the 21 patients met criteria for mixed dementia, indicating cognitive decline attributable to both AD and vascular pathology. Seven patients received anodal tDCS targeting the left DLPFC (2 mA, 20 min per session) for four consecutive days, while five patients received sham stimulation. Visual recall, reaction times in the n-back task, and performance in the go/no-go test improved only in the active tDCS group, suggesting that tDCS may facilitate working memory and executive function rehabilitation.

Gangemi et al. (2024) conducted a controlled pilot study to examine the effects of tDCS on neurophysiological and cognitive outcomes in 30 patients with VaD. Neurophysiological outcomes were assessed using P300 event-related potentials, and cognitive performance was evaluated with the MMSE. Compared with controls, the tDCS group exhibited reduced P300 latency, increased P300 amplitude, and significant improvements in MMSE scores, indicating faster cognitive processing, stronger neural responses, and overall cognitive enhancement.

More recently, Cao et al. (2026) conducted an open-label pilot study to examine High-Definition Transcranial Direct Current Stimulation (HD-tDCS) in patients with CSVD. HD-tDCS is a refined form of tDCS that typically uses multiple small electrodes to achieve more focal and targeted modulation of cortical regions than conventional tDCS. In the study, 20 patients with CSVD received 14 days of HD-tDCS targeting the left DLPFC. After treatment, improvements were observed in global cognition, memory function, information-processing speed, and attention, accompanied by decreased resting-state functional connectivity between the hippocampal cognitive subregion and the left dorsolateral superior frontal gyrus. These findings suggest that HD-tDCS may improve cognitive function in CSVD by modulating hippocampal-prefrontal network connectivity, although the open-label design, small interventional sample, and lack of sham control limit causal interpretation.

Methodological characteristics and key limitations of studies investigating tDCS in non-stroke VCI were listed in Table 4.

**Table 4:** Study characteristics and key limitations of included studies on tDCS in non-stroke VCI.

Author, year	Study design	Sample size	Blinding	Control	Key limitations
André et al. (2016)	RCT	*n* = 21 (2 groups: 13/8)	Single-blind	Sham	Short intervention duration; no concurrent cognitive training.
Gangemi et al. (2024)	Pilot quasi-randomized controlled trial	*n* = 30 (2 groups: 15/15)	Double-blind	Sham	Lack of adequate randomization; no concurrent cognitive training.
Cao et al. (2026)	Open-label pilot study	*n* = 20	No blinding	No control	No blinding and control; very small sample size.

## 4 Discussion

This review examined the current evidence for tDCS-based cognitive rehabilitation in patients with VCI. Overall, most included studies have focused on the effects of tDCS in patients with PSCI, and several reported improvements in global cognition, executive function, or other cognitive outcomes after repeated stimulation sessions. In contrast, only three studies examined tDCS in non-stroke VCI populations. While tDCS shows favorable effects on cognitive function, it is important to note that most available evidence is derived from PSCI populations. The included studies also exhibited substantial heterogeneity in patient characteristics, lesion profiles, stimulation targets, stimulation intensity, intervention duration, concomitant rehabilitation strategies, and cognitive outcome measures. This variability limits direct comparability across studies and may partly explain the inconsistency of reported effects.

### 4.1 Variability in stimulation parameters and protocols

Most included tDCS studies applied stimulation over the DLPFC, particularly the left DLPFC region. This target is theoretically relevant because the DLPFC is a key node of the frontoparietal control network and is involved in working memory, cognitive control, attention regulation, and executive function. These domains are frequently affected in PSCI, providing a plausible rationale for selecting the DLPFC as a stimulation target. In the included studies, DLPFC-targeted protocols more frequently reported improvements in global cognition and executive function, which is consistent with its role in higher-order cognitive processes (Mancuso et al., 2016). However, the predominance of left DLPFC stimulation may also partly reflect conventions derived from tDCS studies in mild cognitive impairment, AD, and experimental cognitive neuroscience. Given the heterogeneity of lesion location, lesion laterality, cognitive profiles, and network disruption after stroke, a standardized left DLPFC montage may not be optimal for all PSCI patients. Other cortical targets, including the parietal regions, temporal regions, and the motor cortex, were also explored in selected studies, suggesting that stimulation targets may need to be matched to the dominant cognitive deficit and underlying network disruption (Yun et al., 2015; Kim et al., 2019; Hu et al., 2023).

Across tDCS studies, relatively low intensities (1–2 mA) were typically applied, which were less likely to cause discomfort while remaining sufficient to modulate neuronal excitability. However, intervention schedules varied substantially, including 3, 5, or 7 sessions per week over 1 to 8 weeks. Descriptively, studies with longer intervention durations and higher session frequency appeared more likely to report cognitive improvements, although this pattern was not consistent and cannot be interpreted as evidence of a dose–response relationship. Moreover, the clinical utility of tDCS depends not only on immediate post-intervention cognitive improvement but also on whether these gains are durable. Most included studies assessed outcomes shortly after the intervention, and only a limited number incorporated post-treatment follow-up (Ko et al., 2022; Ai et al., 2024). In these studies, the cognitive differences observed between the stimulation and control groups did not appear to substantially change during the follow-up period, suggesting that the treatment-related effects may have been maintained over the short term. However, because follow-up data were available in only a limited number of studies and the duration of follow-up was generally short, the long-term durability of tDCS-induced cognitive benefits remains insufficiently established.

Overall, the variability in stimulation targets and cumulative dose limits direct comparison across studies and may contribute to the inconsistency in reported cognitive effects, highlighting the need for future studies to systematically evaluate the influence of stimulation parameters on cognitive functions in VCI.

### 4.2 Imaging evidence of possible neurophysiological mechanisms

Although standard cognitive assessments such as the MoCA and MMSE were commonly used, cognitive rehabilitation often progresses gradually, becoming detectable only after substantial improvements have accumulated. In addition, the underlying mechanisms of tDCS remain insufficiently understood and require further research. To address this, several studies have employed neuroimaging and electrophysiological techniques, including fNIRS, EEG and BOLD-fMRI, to explore potential alterations in brain oscillations and networks (Chu et al., 2022; Wang et al., 2022; Yang et al., 2022a; Hu et al., 2023). These studies suggest that tDCS may be associated with modulation of cortical excitability, neural oscillatory activity, and functional connectivity within prefrontal cognitive control networks. An fMRI-based tDCS study further demonstrated that higher reliability and improved cognitive performance were associated with increased tracking-related activation in the dorsal attention network, default mode network, and anterior cingulate cortex after the intervention compared with baseline (Kolskår et al., 2021). However, the current evidence is still limited and largely derived from small-scale or pilot studies, and therefore should not be interpreted as definitive mechanistic conclusions.

### 4.3 Combined effects of tDCS and cognitive training

The combination of tDCS with cognitive training may enhance cognitive rehabilitation. In most studies, cognitive training served as the primary intervention, while tDCS was applied as an adjunctive method. Results showed that groups receiving combined tDCS and cognitive training achieved greater cognitive improvements than the groups receiving cognitive training alone. Cognitive training approaches were diverse with CCT becoming increasingly prevalent due to its ability to enhance participant engagement and sustain motivation. A meta-analysis of 17 trials reported that CCT produced small to moderate effects on global cognition, working memory, learning, and memory in patients with mild cognitive impairment (Hill et al., 2017). In post-stroke populations, CCT appears to be a suitable approach to improve working memory performance, although further research is needed to evaluate the combined effect of tDCS, given the limited number of studies (Kazinczi et al., 2024).

Beyond the introduction of cognitive training, questions remain regarding whether concurrent application of tDCS and cognitive training offers greater benefits than non-concurrent protocols. Concurrent cognitive training may allow patients to optimize rehabilitation time while simultaneously activating relevant brain areas. The combination of concurrent tDCS and cognitive training may facilitate cognitive recovery by accumulating cortical activation. A factorial design study found that CACT combined with tDCS produced greater improvements in global cognition, subdomains of cognitive function, and activities of daily living compared with CACT or tDCS alone (Chen et al., 2024). Previous studies have suggested that tDCS effects are influenced by baseline cognitive state, task difficulty, and concurrent task engagement (Hsu et al., 2016), and that the interaction between stimulation and task-induced neural activity may be critical for shaping stimulation-induced plasticity (Bortoletto et al., 2015). Therefore, concurrent stimulation may provide a theoretically favorable window for modulating task-relevant networks, but further studies are required to directly compare concurrent and non-concurrent protocols under controlled stimulation and training conditions.

### 4.4 Neurovascular effects of tDCS

Several studies suggest that tDCS may modulate CBF, but the relevance of these findings to VCI, particularly CSVD-related VCI, requires cautious interpretation (Bahr-Hosseini and Bikson, 2021; Bahr-Hosseini et al., 2023). Evidence from stroke populations suggests that cathodal tDCS has been investigated for its potential neuroprotective effects in acute ischemic stroke, possibly involving perfusion-related mechanisms, whereas anodal tDCS has been reported to influence CBF in chronic stroke and hypoperfusion contexts (Gunduz et al., 2024). The regulation of CBF is tightly linked to neuronal activity through neurovascular coupling, in which neurons, glial cells, and vascular components collectively modulate regional perfusion (Yang et al., 2022b). In CSVD, however, this neurovascular unit is often disrupted by endothelial dysfunction, blood–brain barrier impairment, white matter injury, and reduced cerebrovascular reactivity, all of which may alter the vascular response to neuromodulation (Zanon Zotin et al., 2021). Therefore, although tDCS may have the potential to influence CBF and neurovascular regulation, whether such effects contribute to cognitive improvement in CSVD-related VCI remains uncertain and requires direct validation in future studies.

### 4.5 Safety and tolerability

The safety profile of tDCS is an important consideration for its clinical application in VCI populations. Overall, tDCS is generally considered safe and well tolerated when applied within commonly used stimulation parameters, and serious adverse events have rarely been reported in previous safety reviews (Brunoni et al., 2011; Bikson et al., 2016). A safety review focusing on stroke populations also suggested that reported adverse events were generally mild and transient sensations, including tingling, itching, burning sensation, headache, fatigue, dizziness, and local skin redness (Russo et al., 2017). Across the included studies, only two reported mild adverse reactions such as itchy sensation and local skin reddening, and no serious adverse events were reported in the included studies (Ai et al., 2024; Zhang et al., 2024). However, adverse-event reporting was incomplete across the included studies, and the absence of reported serious adverse events should therefore be interpreted cautiously. Safety considerations may be particularly important in PSCI populations because stroke lesions may be associated with potentially modified seizure thresholds, and sensory impairment after stroke may influence patients’ perception of stimulation-related discomfort and compromise sham blinding. Future studies should therefore systematically document seizure history, neurological comorbidities, lesion characteristics, adverse events, and blinding integrity to better evaluate the safety and tolerability of tDCS in PSCI and VCI populations.

### 4.6 Limitations and future directions

Several limitations of this review should be acknowledged. First, as this study was conducted as a structured narrative review, substantial heterogeneity existed across the included studies in terms of participant grouping, stimulation protocols, intervention duration, and outcome measures, which limited the feasibility of performing a systematic quantitative analysis or meta-analysis. In addition, this review was not prospectively registered in a public database, which may reduce methodological transparency and reproducibility. Moreover, due to the substantial imbalance in the available literature, meaningful comparisons between PSCI and non-stroke VCI populations could not be performed. It also remains unclear whether tDCS exerts differential effects in mixed dementia compared with pure vascular dementia, given the scarcity of available evidence. Therefore, the conclusions of this review should be interpreted primarily in the context of tDCS studies in PSCI populations rather than generalized to the entire VCI spectrum.

Even within PSCI populations, differences in lesion characteristics and stroke stage may contribute to heterogeneity in cognitive outcomes and recovery patterns. However, these variables were not consistently reported across the included studies, limiting further interpretation. Furthermore, stroke lesion location, lesion volume, and tissue loss in PSCI participants may alter tissue conductivity and current flow, thereby influencing the distribution of the induced electric field. Future studies would benefit from more detailed lesion characterization and the incorporation of MRI-derived individualized electric-field modeling to optimize stimulation targets and current dosing.

Future tDCS protocols should also be tailored to specific VCI subtypes. Compared with stroke-induced VCI, patients with CSVD typically do not exhibit large-scale brain lesions. Instead, cerebral microvascular endothelial dysfunction, manifested as blood–brain barrier disruption, impaired vasodilation, and abnormal cerebral blood flow (Gorelick et al., 2011; Wardlaw et al., 2019), may contribute to functional impairment in CSVD patients. Evidence suggests that tDCS has the potential to influence neurovascular coupling by inducing vascular responses and modulating interactions among neurons, astrocytes, and vascular components within the neurovascular unit (Bahr-Hosseini and Bikson, 2021; Luo et al., 2022). Future studies directly examining the effects of tDCS on neuroimaging markers, such as WMH burden, CBF, and BBB integrity, in CSVD-related VCI are essential for the development of more targeted stimulation strategies for this population. Genetic markers may also help identify individual differences in neuromodulatory responsiveness and cognitive recovery potential. Moreover, given the diffuse and network-level nature of CSVD pathology, stimulation strategies based solely on a focal left DLPFC montage may not be sufficient for all patients. Future studies should explore whether bilateral prefrontal stimulation, frontoparietal network-level montages, or individualized network-guided stimulation protocols are more appropriate for this population.

Although this review focuses on tDCS, other non-invasive intervention modalities, such as transcranial alternating current stimulation (tACS), may represent potential future directions for cognitive rehabilitation in VCI. Evidence from related post-stroke conditions suggests that tACS may influence oscillation-dependent plasticity and recovery processes in specific syndromes such as aphasia and hemispatial neglect (Schuhmann et al., 2022; Xie et al., 2022). Mechanistically, tACS may be relevant to VCI because it can modulate endogenous neural oscillations and network synchronization related to attention, working memory, and executive control. However, no clinical studies have directly examined tACS for cognitive rehabilitation in VCI populations and its clinical efficacy, optimal targets, and stimulation protocols remain to be determined in future studies.

## 5 Conclusion

Current evidence indicates that tDCS may have potential benefits for cognitive rehabilitation, particularly in patients with PSCI. However, the available evidence remains highly imbalanced, with most studies focusing on PSCI and very limited data available for non-stroke VCI. These potential cognitive benefits may be associated with the modulation of cortical excitability, neural oscillatory activity, and functional connectivity. Preliminary evidence also suggests that tDCS may influence CBF and neurovascular coupling, although these vascular effects remain insufficiently established in VCI populations and should be interpreted cautiously. When paired with cognitive training, tDCS may further enhance rehabilitation outcomes beyond training alone. However, current research is mainly focused on stroke-related VCI, leaving its efficacy in other vascular pathologies insufficiently explored. Further research is needed to clarify both the neural and vascular mechanisms of tDCS to facilitate its wider clinical application in VCI.
