# Correction: Transcranial direct current stimulation to improve cognitive function in patients with vascular cognitive impairment: a literature review

**DOI:** 10.3389/fnagi.2026.1967681

**Published:** 2026-10-21

**Authors:** Jiamian Shi, Xiaodong Li, Yubiao Sun, Ling Guan

**Affiliations:** 1 Department of Biomedical Engineering, Medical Science and Engineering, Beijing Institute of Technology, Beijing, China; 2 School of Interdisciplinary Science, Beijing Institute of Technology, Beijing, China; 3 Department of Neurology, Beijing Tiantan Hospital, Capital Medical University, Beijing, China

**Keywords:** cerebral small vessel disease, noninvasive neuromodulation, stroke, transcranial direct current stimulation, vascular cognitive impairment

In the published article, the corresponding authors were presented in the wrong order in the author list. The correct author order places Yubiao Sun second last and Ling Guan last. The correct author list appears below.

Jiamian Shi^1^, Xiaodong Li^1^, Yubiao Sun^2^^*^ and Ling Guan^3^^*^

To maintain the sequential ordering required by Frontiers, the affiliation numbers and affiliation section have also been corrected to read:

^1^Department of Biomedical Engineering, Medical Science and Engineering, Beijing Institute of Technology, Beijing, China

^2^School of Interdisciplinary Science, Beijing Institute of Technology, Beijing, China

^3^Department of Neurology, Beijing Tiantan Hospital, Capital Medical University, Beijing, China

The original version of this article has been updated.

