# Supplementary material for: Transcranial direct current stimulation to improve cognitive function in patients with vascular cognitive impairment: a literature review
Source: Front Aging Neurosci. 2026 Jul 1;18:1829594. doi: 10.3389/fnagi.2026.1829594 (PMC13368876; doi:10.3389/fnagi.2026.1829594)
Supplement: Supplementary file 1 [file Table_1.docx]

Supplementary Table S1. Complete database-specific search strategies

| PubMed |  |  |
| --- | --- | --- |
|  | 1 | "tDCS"[tw] OR "Transcranial Direct Current Stimulation"[Mesh] OR ("Transcranial"[tw] AND "Direct"[tw] AND "Current"[tw] AND "Stimulation"[tw]) |
|  | 2 | ("vascular"[tw] AND (("cognitive"[tw] AND ("impairment*"[tw] OR "dysfunction"[tw] OR "disorder"[tw])) OR "Cognitive Dysfunction"[Mesh] OR "Dementia"[MeSH] OR "dementia"[tw])) |
|  | 3 | "stroke"[tw] AND (("cognitive"[tw] AND ("impairment*"[tw] OR "dysfunction"[tw] OR "disorder*"[tw])) OR "Cognitive Dysfunction"[MeSH] OR "dementia"[MeSH] OR "dementia"[tw] OR ("mixed"[tw] AND "dementia"[tw])) |
|  | Final search string | #1 AND (#2 OR #3) NOT "Review"[Publication Type] NOT ("animals"[MeSH] NOT "Humans"[MeSH]) |
| WOS | TS=(("tDCS" OR "transcranial direct current stimulation" OR ("transcranial" AND "direct" AND "current" AND "stimulation")) AND ((vascular AND ((cognitive AND (impairment* OR dysfunction OR disorder*)) OR dementia)) OR (stroke AND ((cognitive AND (impairment* OR dysfunction OR disorder*)) OR dementia OR "mixed dementia")))) | |
| Scopus | TITLE-ABS-KEY(  (  " tDCS "  OR "transcranial direct current stimulation"  OR ("transcranial" AND "direct" AND "current" AND "stimulation")  )  AND  (  (vascular AND ((cognitive AND (impairment* OR dysfunction OR disorder)) OR dementia))  OR  (stroke AND ((cognitive AND (impairment* OR dysfunction OR disorder*)) OR dementia OR ("mixed" AND "dementia")))  )  ) | |

In Web of Science (WOS) and Scopus, reviews were excluded using database-specific document type filters after the initial search, and animal-only studies were further excluded during title/abstract and full-text screening.
